# The Renewel of Life

**Published:** 1865-09

**Authors:** 


THE RENEWAL OF LIFE. LECTURES, CHIEFLY CLINICAL. By THOS. KING CHAMBERS, M.D., Honorary Physician to H. R. H. the Prince of Wales; Physician to St. Mary's and the Lock Hospitals. From the third London edition. Philadelphia: LINDSAY & BLAKISTON. 1865.

This is an elegantly published octavo volume, of 638 pages; containing 52 lectures, on the following topics:—Life and death; disease and cure; formation of mucus and pus; typh.-fever; small-pox; rheumatic fever; gonorrhœal rheumatism; pericarditis; pleurisy; hydrothorax; acute laryngitis; capillary catarrh; pneumonia; emphysema of lungs; pulmonary consumption; thoracic aneurism; disease of the heart; purpura; anæmia; prominence of eyeballs; atrophy of muscles; chorea; epilepsy; hysteria; spinal paralysis; sciatica; albuminuria; ascites; diabetes; mortification; importance of the digestive organs in therapeutics; indigestion in general; slow digestion and acidity; pain in the stomach; eructation and vomiting; diarrhœa; costiveness and constipation; dietetics; corpulence; on pepsin; on alcohol; on blood-letting; answers to objections.

The reader will find much in this book that is valuable, mixed with some doctrines which we deem, not only erroneous, but decidedly mischievous in their influence. We shall notice some of the chapters more in detail at a future time.

